# The road to recovery: an interrupted time series analysis of policy intervention to restore essential health services in Mexico during the COVID-19 pandemic

**DOI:** 10.7189/jogh.12.05033

**Published:** 2022-07-23

**Authors:** Svetlana V Doubova, Catherine Arsenault, Saul E Contreras-Sánchez, Gabriela Borrayo-Sánchez, Hannah H Leslie

**Affiliations:** 1 Epidemiology and Health Services Research Unit CMN Siglo XXI, Mexican Institute of Social Security, Mexico City, Mexico; 2 Department of Global Health and Population, Harvard TH Chan School of Public Health, Boston, USA; 3 Epidemiology and Health Services Research Unit CMN Siglo XXI, Mexican Institute of Social Security, Mexico City, Mexico; 4 Coordinación de Innovación en Salud, Instituto Mexicano del Seguro Social, Ciudad de México, México; 5 Division of Prevention Science, University of California San Francisco, San Francisco, CA< USA

## Abstract

**Background:**

Recovery of health services disrupted by the COVID-19 pandemic represents a significant challenge in low- and middle-income countries. In April 2021, the Mexican Institute of Social Security (IMSS), which provides health care to 68.5 million people, launched the National Strategy for Health Services Recovery (Recovery policy). The study objective was to evaluate whether the Recovery policy addressed COVID-related declines in maternal, child health, and non-communicable diseases (NCDs) services.

**Methods:**

We analysed the data of 35 IMSS delegations from January 2019 to November 2021 on contraceptive visits, antenatal care consultations, deliveries, caesarean sections, sick children’s consultations, child vaccination, breast and cervical cancer screening, diabetes and hypertension consultations, and control. We focused on the period before (April 2020 – March 2021) and during (April 2021 – November 2021) the Recovery policy and used an interrupted time series design and Poisson Generalized Estimating Equation models to estimate the association of this policy with service use and outcomes and change in their trends.

**Results:**

Despite the third wave of the pandemic in 2021, service utilization increased in the Recovery period, reaching (at minimum) 49% of pre-pandemic levels for sick children’s consultations and (at maximum) 106% of pre-pandemic levels for breast cancer screenings. Evidence for the Recovery policy role was mixed: the policy was associated with increased facility deliveries (IRR = 1.15, 95%CI = 1.11-1.19) with a growing trend over time (IRR = 1.04, 95%CI = 1.03-1.05); antenatal care and child health services saw strong level effects but decrease over time. Additionally, the Recovery policy was associated with diabetes and hypertension control. Services recovery varied across delegations.

**Conclusions:**

Health service utilization and NCDs control demonstrated important gains in 2021, but evidence suggests the policy had inconsistent effects across services and decreasing impact over time. Further efforts to strengthen essential health services and ensure consistent recovery across delegations are warranted.

The COVID-19 pandemic has put enormous stress on health systems globally. The first year of the pandemic saw disruptions and constraints on routine health services in many countries due to COVID-19 containment measures, prioritization of COVID-19 health care, and population reluctance to seek services out of concern over exposure, and other causes [1,2]. The disruptions in routine preventive, screening, and curative services generated significant backlogs in care and increased population risk for preventable morbidity and mortality [1,2]. In 2020, multiple countries faced excess mortality that was higher than COVID-19-related fatalities, pointing to substantial indirect mortality [3]. Numerous low- and middle-income countries (LMICs) reported increases in maternal, child, and non-communicable diseases (NCD) related mortality [4-6].

Prompt recovery of essential health services is imperative to meet the health-related sustainable development goals and improve population health [4]. This is a significant challenge in LMICs, where health resource constraints were exacerbated by the COVID-19 pandemic. Essential services recovery requires additional funding and a comprehensive strategy to reactivate interrupted services and overcome backlogs in care [7].

The Mexican Institute of Social Security (IMSS) is Mexico's largest national public health care institution. IMSS delivers health care to workers employed in the formal labour market and their families ( ~ 68.5 million people), representing more than half of Mexico’s population. IMSS provides health care through its nationwide network of primary, secondary, and tertiary health care facilities [8]. Between March and May 2020, IMSS implemented an organized COVID-19 response following Ministry of Health recommendations. The major planks of this strategy included allocating facilities, health workers and funding towards COVID-19 care, reducing routine service use by decreasing routine health appointments and making limited services available virtually, and contracting out deliveries and emergency services to private hospitals [8]. The resource redistribution towards COVID-19 care and the strategies to reduce congestion in health facilities resulted in a substantial decline in essential health services provision. During the first nine months of the pandemic, from April to December 2020, across five maternal and child services and four chronic care services, an estimated total of 8.74 million patient visits were lost [9]. Breast and cervical cancer screening, sick child visits, and contraceptive services declined by more than half, vaccinations declined by 36%, while the number of diabetes, hypertension and antenatal care consultations declined by one-third [9].

To address this decline, IMSS launched the aforementioned Recovery policy in April 2021, comprising six main components: 1) reconversion of previously repurposed COVID-19 hospitals back to routine care; 2) strengthening COVID-19 preventive measures; 3) adjusting essential health services governance, optimizing service delivery and organizing weekend health services; 4) implementation of telemedicine services, including virtual consultations for individuals with controlled chronic diseases in select clinics; 5) strengthening of preventive services and health promotion activities, and 6) essential health services monitoring [10]. This study aims to evaluate if the Recovery policy addressed COVID-19-related decline in reproductive, maternal, child health (RMCH) and NCDs services and to quantify the policy’s impact on service delivery in the ongoing pandemic.

## METHODS

We conducted a secondary data analysis of the IMSS health information system data.

### Data source

Data for this study were available at the delegation level. We used monthly data from the IMSS health information system (HIS) for the period of January 2019 to November 2021 (35 months) based on data available by January 2022. This included 15 months before (January 2019 to March 2020) and 20 months during (April 2020 to November 2021) the COVID-19 pandemic. IMSS has 1523 primary health care facilities and 283 hospitals located in 35 IMSS delegations across 32 Mexican states (one delegation per state except in Mexico City, the State of Mexico, and Veracruz, with two delegations per state). Health facilities submit information monthly via the HIS to the Coordination of Information and Strategic Analysis in each delegation and from there to the headquarters of the IMSS Health Information Division in Mexico City. The IMSS Health Information Division conducts regular, systematic validations based on the IMSS technical standards. This process includes checking values for plausibility through data corroboration with delegations or facilities. Prior to analysis, we reviewed data for outliers and confirmed values as relevant. We identified months without data for specific childhood vaccines and confirmed no vaccinations of that type had been administered before setting these months to zero; no data were missing for analysis. The data for patients with diabetes and hypertension included information on both in-person and virtual consultations; the latter consultations for individuals with controlled chronic diseases were offered in 18% of family medicine clinics in 2021.

### Measures

#### Outcomes: service utilization and care outcomes

The study focused on 12 indicators of service use and care outcomes provided at IMSS. These indicators were defined and analysed previously to describe the decline in RMCH and NCD essential health services during the first nine months of the COVID-19 pandemic (Table S1 in the **Online Supplementary Document**) [9]. Briefly, child health care included the number of consultations for children under five with diarrhoea, pneumonia, or malnutrition in primary care clinics, and the total number of children who completed the final required dose of the following vaccines: Bacillus Calmette-Guérin (BCG) vaccine for children under 1 and the rotavirus vaccine for children under 10; pentavalent vaccine against diphtheria, tetanus, pertussis, polio and *Haemophilus influenzae* type B; the pneumococcal vaccine; and the measles, mumps and rubella (MMR) vaccine. Vaccination service use was aggregated into one indicator for the main analysis. Reproductive and maternal health indicators comprised the number of reproductive-age women who used contraceptive services, number of antenatal care consultations, number of facility deliveries, and (as a care outcome) the number of caesarean section (C-section) deliveries. Unlike other indicators, increases in C-section deliveries would indicate poor quality care, given the very high baseline rate of C-sections and the high level (55%) of C-sections performed on pregnant women in Mexico without complications [11]. Within chronic disease services, we considered preventive services for cancer screenings (the number of women aged 25 to 64 screened for cervical cancer (Papanicolaou test) and of women aged 50 to 69 screened for breast cancer (mammography)) as well as disease management (the number of consultations for diabetes and hypertension among adults aged 20 or older at primary care clinics). For care outcomes, we calculated the number of patients with diabetes and hypertension who had a controlled condition: fasting blood glucose tests 70-130 mg/dl among patients with diabetes and blood pressure tests <140/90mmHg among patients with hypertension. These indicators of service utilization (absolute number of consultations) and care outcomes (C-sections and disease control) span the most common reasons for consultations at IMSS [8,9].

#### Exposure: Recovery policy

We defined the policy intervention of interest, the Recovery policy, as effective from April 2021 onwards, and we modelled this intervention as potentially having immediate effects (level change) as well as incremental effects over time (slope change). We considered observed data within three periods: pre-pandemic (January 2019 to March 2020, 15 months), pandemic pre-policy period (April 2020 to March 2021, 12 months) and pandemic policy period (April 2021 to November 2021, 8 months).

#### Time-varying covariates

To adjust for health service context during this period, we defined a covariate for months since the start of the pandemic. To adjust for secular trends in analyses including pre-COVID-19 data, we defined a covariate for months since January 2019. We included indicator terms for seasons to capture seasonality of health service need and utilization. Finally, to capture the severity of the COVID-19 pandemic at each point in time, we calculated the average number of IMSS inpatient beds occupied by COVID-19 patients per month for each delegation across the analytic period. We use the natural log of occupied beds based on the skewed distribution of this variable.

### Statistical analysis

The original analytic plan for this study was registered with IMSS in September 2021; however, we updated the plan based on additional data available online. To describe service volume before the pandemic and during the pre-Recovery and Recovery periods, we averaged visits over the months within each analytic period by delegation and report service volume for median delegation and first and third quartiles. We plotted observed data for national visit numbers for each month of these periods and used flexible fractional polynomial fitted lines to illustrate trends.

To estimate changes in service delivery during the Recovery period, we focused on the two periods during the pandemic (April 2020 onwards) and used an interrupted time-series design, modelling the Recovery policy by including a level change for the overall pandemic policy period and a slope change for each month elapsed during the policy period.

For the primary analysis, we used generalized estimating equation (GEE) Poisson regression models, defining the exposed population as the number of IMSS affiliates per delegation during 2021 in the following population groups: 1) children under 5: sick child visits; 2) children under 10: vaccines; 3) women aged 15-49 years: contraceptive use, antenatal care use, deliveries; 4) women aged 25-64 years: cervical cancer screening; 5) women aged 50-69 years: breast cancer screening; 6) total deliveries: C-sections performed; 7) adults aged 20+ years: hypertension visits, diabetes visits; 8) diabetes visits: patients with controlled diabetes; 9) hypertension visits: patients with controlled hypertension. We checked the distribution of variables such as beds occupied by COVID-19 patients and considered transformations that may enhance the interpretability and meaning of these variables. We modelled the potential impact of the Recovery policy on service utilization without a lag, assuming the policy could affect service utilization in real time. We included a 1-month lag for disease control outcomes (hypertension control, diabetes control) on the hypothesis that resumption in clinical care would affect health outcomes after 1 month. We selected the correlation structure that best fit the data by visually inspecting the correlation structure for serial correlation using variograms and calculating the quasi-likelihood under the independence model criterion (QIC). We compared QIC for first-order auto-regressive correlation and exchangeable correlation and selected the option that minimized QIC: auto-regressive for child health services and non-communicable disease service utilization outcomes, exchangeable for reproductive health outcomes and disease control outcomes.

To quantify Recovery policy impact, we compared observed outcomes to predictions based on pre-COVID-19 trends and pre-Recovery policy trends. To compare observed outcomes to levels of service delivery expected in the absence of the COVID-19 pandemic, we fit Poisson regression models on data from the pre-pandemic period, controlling for elapsed months and season, and predicted service volume for November 2021 assuming pre-pandemic trends had continued. To compare to the counterfactual scenario of no Recovery policy, we predicted outcomes for each delegation in November 2021 for each service analysed using the main models described above, but setting Recovery policy to 0 throughout this period. We compared observed visits to the 95% confidence interval for predicted visits per delegation under the counterfactual scenario of no Recovery policy; we totalled the observed and predicted visits across delegations to provide a national summary.

### Sensitivity analyses

We conducted sensitivity analyses to test the robustness of our analytic findings. First, to address potential differences in care-seeking and service delivery mechanisms by childhood age, we analysed the childhood vaccines individually, using the exposure population as children under 1 for BCG and children under 10 for all other vaccines. Second, to calculate Recovery policy effects as a difference in visit numbers, we fit linear GEE models with an identity link and the best fitting correlation structure based on QIC as above. Third, to test the robustness of the findings against the assumption of immediate policy effects, we added a 1-month lag to the policy covariates (making it a 2-month lag for disease control outcomes) to assess differences in outcomes under an alternative assumption that the Recovery policy took 1 month to be fully implemented and to affect patient behaviours, in keeping with existing studies on population responses to COVID-19 policies [12].

All analyses were conducted using STATA version 16 (Stata Corp, College Station, TX, USA). The STATA code was cross-checked within the research team.

## RESULTS

The data set included 1225 observations for each service across the 35 delegations spanning 35 months. These observations reflect 107.25 million patient visits for nine RMCH and NCD services provided from January 2019 to November 2021.

**Figure 1:**
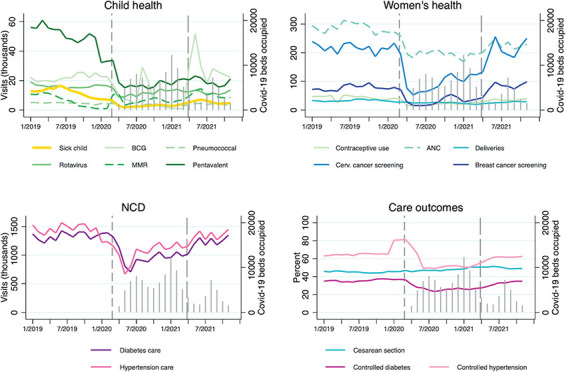
Trends in health service use and outcomes, Mexican Institute of Social Security, January 2019 – November 2021. Lines provide observed data totalled across delegations; grey spikes show the number of patients admitted with COVID-19 to IMSS, and dashed vertical lines demarcate the start of the pandemic (April 2020) and the implementation of the Recovery policy (April 2021). Indicator definitions are in Table S1 in the **Online Supplementary Document**. Controlled diabetes and hypertension are among those who visited a primary care facility for a consultation only.

From the initial pandemic response in April 2020 to the Recovery policy in April 2021, Mexico experienced two major waves of COVID-19 cases and hospitalizations, peaking in July 2020 with over 8000 individuals hospitalized at IMSS and again in January 2021 with over 12000 hospitalizations. A third wave during the Recovery policy peaked in August 2021 with over 7000 hospitalizations. **Figure 1** shows the service utilization and care outcomes from January 2019 to November 2021 and the underlying COVID-19 burden at the national level. Several indicators were in flux pre-pandemic, including declines in child health services and increases in hypertension control. Declines in service volume and care outcomes aside from C-sections were notable during the early pandemic period, with uneven recovery prior to and following the Recovery policy. As detailed in **Table 1** using median levels across delegations, all service volumes showed substantial declines in the first year of the COVID-19 pandemic, ranging from 24% of pre-pandemic service volume for consultations for sick children to 78% for facility deliveries; caesarean section rate remained unchanged, but disease control decreased for both diabetes (70% of pre-pandemic levels) and hypertension (83%). While all services increased during the Recovery policy period, cancer screenings approached pre-pandemic levels but consultations for sick children and contraceptive use visits remained less than two-thirds of pre-pandemic volume.

**Table 1:** Health service delivery from January 2019 to November 2021, Mexican Institute of Social Security*

	Pre-COVID-19 period, January 2019 – March 2020, N = 35 delegations	COVID-19 period before Recovery policy, April 2020 – March 2021, N = 35 delegations		COVID-19 Recovery policy period, April 2021 – November 2021, N = 35 delegations	
Indicator	**Median delegation (Q1, Q3)**	**Median delegation (Q1, Q3)**	**% of pre-pandemic level**	**Median delegation (Q1, Q3)**	**% of pre-pandemic level**
**Child health care:**					
Consultations for sick children†	312 (196, 416)	74 (51, 100)	24%	152 (91, 207)	49%
Childhood vaccinations administered‡	2421 (1249, 3191)	1121 (592, 1750)	46%	1856 (896, 2557)	77%
**Reproductive and maternal health care:**					
Contraceptive use visits	984 (581, 1613)	391 (225, 862)	40%	631 (462, 1458)	64%
Antenatal care visits	6634 (3864, 10 279)	4 304 (2730, 6 877)	65%	5 040 (3021, 7 967)	76%
Facility deliveries (incl. caesarean sections)	708 (428, 1420)	552 (304, 1005)	78%	626 (388, 1123)	88%
Caesarean section rate (%)	48 (41, 51)	49 (45, 54)	102%	52 (46, 56)	108%
**Cancer screenings:**					
Women screened for cervical cancer§	4112 (2269, 8620)	1728 (833, 3523)	42%	3990 (2083, 8581)	97%
Women screened for breast cancer	1357 (878, 3077)	579 (402, 940)	43%	1432 (975, 3120)	106%
**Noncommunicable disease care:**					
Consultations for diabetes care	34 529 (16 504, 50 009)	24 756 (12 205, 33 200)	72%	30 790 (15 476, 43 205)	89%
Patients with controlled diabetes (%)	33 (29, 40)	23 (19, 30)	70%	29 (25, 35)	88%
Consultations for hypertension care	33 597 (17 718, 60 906)	24 810 (13 285, 45 565)	74%	30 324 (16 901, 57 078)	90%
Patients with controlled hypertension (%)	69 (65, 75)	57 (50, 62)	83%	64 (59, 69)	93%

*Detailed indicator definitions are in Table S1 in the **Online Supplementary Document.**

†Includes consultations for diarrhoea, pneumonia, and malnutrition in children under 5.

‡Number of children who received a unique dose of BCG vaccine, a third dose of pentavalent vaccine, a second dose of measles, mumps and rubella vaccine, a second dose of rotavirus vaccine and a third dose of the pneumococcal conjugate vaccine.

§Number of women aged 25-64 affiliated with IMSS screened with VIA for cervical cancer for the first time.

¶Number of women aged 50-69 affiliated with IMSS screened for breast cancer with a mammography for the first time.

**Figure 2:**
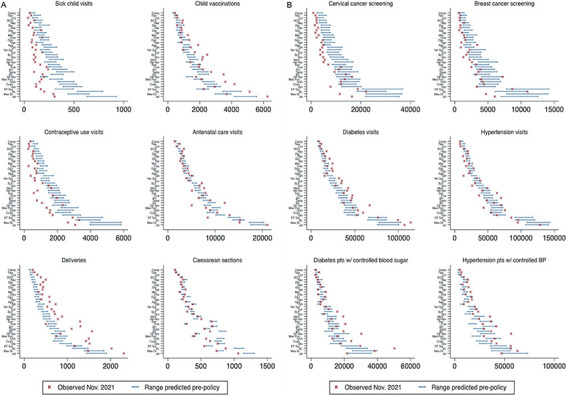
Predicted vs observed service outcomes for November 2021, delegation level. Panel A. Child health services, reproductive and maternal health services. Panel B. Cancer screenings and non-communicable disease care. Delegations are listed on the y-axis in order of population size: Ags – Aguascalientes, BC – Baja California, BCS – Baja California Sur, Camp – Campeche, Chis – Chiapas, Chih – Chihuahua, Coah – Coahuila, Col – Colima, DF Nor – D.F. Norte (Mexico City North), DF Sur – D.F. Sur (Mexico City South), Dgo – Durango, Gto – Guanajuato, Gro – Guerrero, Hgo – Hidalgo, Jal – Jalisco, Mich – Michoacán, Mor – Morelos, Mex Or – México Oriente, Mex Pon – México Poniente, Nay – Nayarit, NL – Nuevo León, Oax – Oaxaca, Pue – Puebla, Qro – Querétaro, QR – Quintana Roo, SLP – San Luis Potosí, Sin – Sinaloa, Son – Sonora, Tab – Tabasco, Tamp – Tamaulipas, Tlax – Tlaxcala, Ver Nor – Veracruz Norte, Ver Sur – Veracruz Sur, Yuc – Yucatán, Zac – Zacatecas.

Primary analysis of the Recovery policy provided mixed evidence of policy effect (**Table 2**). Model findings reflect the overall (level) effect during the policy period and the slope (trend) across the period. For example, the incidence of consultations for sick children was 1.2 times higher during the pandemic policy period (95% CI = 1.12-1.29) but declined by 0.89 per month (95% CI = 0.87-0.92). Except for contraceptive use visits, child health services and reproductive and maternal health services showed statistically significant increases associated with the Recovery policy, although the slope coefficient is negative for all services except for delivery, indicating a declining trend over the study period. Within cancer screenings and NCD consultations, service utilization did not show significant increases attributable to the Recovery policy overall, and slope coefficients indicate discordant trend effects (decreasing for cervical cancer screenings and increasing for diabetes visits). The Recovery policy period was associated with a higher overall incidence of controlled disease among recipients of services diabetes (IRR = 1.05, 95% CI = 1.02-1.08) and hypertension (IRR = 1.09, 95% CI = 1.07-1.11) over time. Based on these models, **Figure 2** shows the observed service levels as of November 2021 compared to the predicted range for each delegation had there been no Recovery policy. For services including delivery, diabetes visits, and hypertension control, observed levels exceed predicted levels in the absence of the Recovery policy, indicating significant differences across most delegations associated with the Recovery policy. Vaccination services and controlled diabetes show significantly more visits than predicted in some but not all delegations, while the remaining services reflect no notable impact of the Recovery policy or even fewer visits than would be expected had early increases in service delivery persisted.

**Table 2:** Poisson regression models of Recovery policy on health service use and outcomes April 2020 – November 2021, Mexican Institute of Social Security (N = 700 delegation months)*

	Level effect of the Recovery policy	Slope effect of the Recovery policy per month
**Indicator**	**IRR (95% CI)**	**IRR (95% CI)**
**Child health care**		
Consultations for sick children	1.20 (1.12-1.29)	0.89 (0.87-0.92)
Childhood vaccinations	1.65 (1.48-1.84)	0.96 (0.95-0.98)
**Reproductive and maternal health care**		
Contraceptive use visits	1.01 (0.95-1.08)	0.94 (0.93-0.96)
Antenatal care visits	1.05 (1.03-1.08)	0.99 (0.98-1.00)
Deliveries (includes caesarean sections)	1.15 (1.11-1.19)	1.04 (1.03-1.05)
Caesarean section deliveries numbers	1.01 (0.99-1.03)	0.99 (0.98-0.99)
**Cancer screenings**		
Cervical cancer screening	0.98 (0.94-1.02)	0.95 (0.93-0.98)
Breast cancer screening	1.06 (0.97-1.16)	0.96 (0.92-1.00)
**Noncommunicable disease care**		
Diabetes visits	0.99 (0.97-1.00)	1.03 (1.01-1.04)
Hypertension visits	0.99 (0.98-1.01)	0.99 (0.98-1.00)
Patients with controlled diabetes	1.05 (1.02-1.08)	1.03 (1.02-1.04)
Patients with controlled hypertension	1.09 (1.07-1.11)	1.01 (1.01-1.02)

CI – confidence interval, IRR – incidence rate ratio

*Poisson models include data from April 2020 to November 2021 (COVID-19 period). Models are adjusted for months since the start of the COVID-19 pandemic, average number of beds occupied by COVID-19 patients per month (log transformed), and seasonality. Population exposure is defined as the 2021 IMSS population per delegation, limited as follows: women aged 15-49 y for contraceptive visits, antenatal care visits, deliveries, total deliveries for cesarean deliveries, children under 5 y for sick child visits, children under 10 y for vaccinations, women aged 25-64 y for cervical cancer screening, women aged 50-69 y for breast cancer screening, adults over 20 for diabetes and hypertension consultations, and consultations for diabetes or consultations for hypertension for control of each disease. For analysis of the quality indicators, the start of the COVID-19 period (starting April 2020) and policy period (starting April 2021) were lagged for one month (May 2020 and May 2021 respectively).

**Table 3** shows this comparison aggregated to the national level and includes predicted visits for November 2021, assuming pre-COVID-19 trends had continued. By November 2021, service volume was at least 90% of expected levels based on pre-COVID trends for all utilization indicators; for disease control outcomes, diabetes control exceeded expected levels while hypertension control lagged at 64% of (very high) expected levels. The observed level of services that appeared to show little positive effect of the Recovery policy by November 2021 (contraceptive use, consultations for sick children, cancer screening services) exceed predictions based on pre-COVID-19 trends (**Figure 2**, Panel A and Panel B). The rebound from deep declines before and at the start of the pandemic may have been observed in the absence of specific policy efforts.

**Table 3:** Observed visits compared to predicted visits for November 2021 based on trends pre-Covid and trends pre-recovery policy, national level

Indicator	Observed	Predicted based on pre-COVID-19 trends*	Predicted based on pre-policy trends†	Observed predicted pre-COVID-19	Observed predicted pre-policy
Consultations for sick children	4664	3081	9767	151%	48%
Childhood vaccinations	68 888	46 147	54 689	149%	126%
Contraceptive use visits	40 056	27 603	62 283	145%	64%
Antenatal care visits	229 643	246 061	233 402	93%	98%
Deliveries (includes caesarean sections)	29 202	31 525	19 660	93%	149%
Caesarean sections	14 290	12 565	15 561	114%	92%
Cervical cancer screening	250 456	181 242	386 964	138%	65%
Breast cancer screening	98 734	95 497	131 035	103%	75%
Diabetes visits	1 348 288	1 482 569	1 112 562	91%	121%
Hypertension visits	1 450 631	972 365	1 548 339	149%	94%
Patients with controlled diabetes (% of observed visits)	34.7	38.8	28.5	90%	122%
Patients with controlled hypertension (% of observed visits)	62.4	97.6	49.1	64%	127%

*National total for November 2021 based on Poisson regression models of observed data January 2019 – March 2020 adjusting for months elapsed and seasonality.

†National total for November 2021 based on Poisson regression models from **Table 2**, setting Recovery policy covariate and months into recovery covariate to equal 0 (counterfactual scenario of no recovery policy).

Sensitivity analyses extended our findings. Analysis of individual vaccines (Tables S2 and S3 in the **Online Supplementary Document**) indicates differential impact across vaccines: incidence was higher during the Recovery policy period most notably for BCG (IRR = 8.83) and modestly for MMR (IRR = 1.29) and pneumococcal vaccines (IRR = 1.12) in adjusted models, with positive trends for BCG but declining trends for pentavalent, MMR, and pneumococcal vaccines as the Recovery policy continued.

Main analysis results on overall Recovery policy impact were sensitive to model specifications. All services except for caesarean section rates showed a positive and significant association with the Recovery policy assuming a 1-month lag for policy implementation (Table S4 in the **Online Supplementary Document**). Trend effect estimates were largely similar to the main analysis: increasing for deliveries, diabetes visits and controlled diabetes, and null for controlled hypertension, but otherwise showing diminishing returns over time.

## DISCUSSION

This study is one of the first to analyse the effectiveness of recovery efforts following the profound effects of the COVID-19 pandemic on health service delivery. Our analysis of 35 months of data from the IMSS information system found mixed evidence of Recovery policy impact, with increased service utilization for maternal and child health services associated with the Recovery policy period as well as gains in disease control for diabetes and hypertension. Our main analysis found no effect on increases in reproductive health services, cancer screenings, and noncommunicable disease visits, although sensitivity analyses assuming a 1-month lag do support initial increases in these services. All findings supported the temporary nature of gains, with only delivery services and BCG vaccination demonstrating sustained increase. The magnitude of change also varied between delegations within the country. The findings suggest the Recovery policy showed some effectiveness, including a gradual resumption of maternal health services and a major recovery in BCG vaccination in particular, but that further efforts are required to strengthen recovery efforts and ensure their effect across services and across delegations in Mexico.

One challenge for health policymakers addressing pandemic-related disruption is defining successful service resumption given the varying trends in health services before the pandemic, the effect of the pandemic itself on demand for health services, and the need to not just resume service delivery, but address the missed visits and backlogs created during the disruption. We compared observed service levels against predictions based on pre-COVID-19 trends and on early pandemic trends following the initial dramatic decline in services. Most services had recovered to at least 90% of levels expected without COVID-19 disruptions by November 2021, although observed service levels fell short of predictions based on the pandemic period for services that saw early rapid recovery. While not definitive, our findings suggest that only delivery services and BCG vaccination for newborns may be on a path toward full recovery and that further policy efforts to bolster routine primary care visits and address the incomplete or waning impact of the initial Recovery policy are warranted. Our findings on care outcomes are more promising, with no evidence of an increase in C-section rates despite delivery volume gains and evidence that the Recovery policy period was associated with a greater incidence of disease control among patients with diabetes or hypertension, gains that continued to increase throughout the policy period. Further assessment of delegations with weaker performance on these outcomes could help target additional recovery efforts. Recovery policies should focus on the evaluation of NCDs care quality and implementation of quality improvement interventions, as previous studies found important gaps in the quality of care delivered to patients with diabetes and hypertension [13-16] that could explain inconsistencies across delegations.

Policy priorities should include the services that saw the least gains relative to pre-pandemic levels (contraceptive use and consultations for sick children) and services that saw steep increases before the Recovery policy but unclear evidence of sustained gains even with the policy (cancer screenings). A temporary relaxation of the screening frequency for the general population and prioritization of the targeted screening of women at high-risk of these cancers based on the age, family history, time since the previous screening, and residence in high deprivation areas were suggested as an effective way for screening services to recover following disruptions related to the COVID-19 pandemic [17].

Our results could be explained in several ways. First, the Recovery policy was implemented during the ongoing COVID-19 pandemic surges in Mexico, with the third pandemic wave occurring from August to September of 2021 as the Recovery policy was under way. This situation has placed a continuous COVID-19 burden on IMSS that may have dampened or undermined policy impacts. It also contributed to decreased demand for services due to the fear of contagion among IMSS affiliates [18]. Additionally, non-pharmacological interventions suggested during the pandemic by the government (eg, social distancing, wearing face masks, using hand sanitisers, and frequent hand washing) could cause a decline in non-COVID-19 related pneumonia and diarrhoea and consequently decrease the need for medical consultations.

Our findings suggest that the effects of the Recovery policy, where identified, largely waned over time; this may reflect an insufficient impact of the policy on overcoming the third pandemic wave, as well as declining service trends dating to before the pandemic, when IMSS affiliates turned to private providers due to long waiting times and health personnel shortages at IMSS [19]. Recovery policy efforts could not address long-standing gaps in the health workforce that predated the pandemic. For instance, IMSS reported 2.3 nurses, 1,4 doctors, and 0.67 beds per 1000 affiliates [8]. In contrast, on average, the Organisation for Economic Co-operation and Development (OECD) member countries had 8.8 nurses, 3.6 physicians, and 4.4 hospital beds [3]. Addressing the health system’s foundations is a critical long-term strategy for strengthening service delivery. Other service delivery innovations, such as continuing weekend working hours or integrating multiple services like childhood vaccination with cancer screening for family members, may be required. As the most recent pandemic wave wanes, public communication about the established safety measures may help to guarantee safe service delivery, address COVID-19-related fears and create trust. Moreover, media and immunization campaigns and phone contacts to schedule clinic or home visits for children with missing vaccination could be useful to catch up on missing immunizations [20].

We conducted an observational interrupted time series analysis to estimate the real-world effectiveness of the Recovery policy on health service utilization and outcomes. In the context of the national policy addressing an ongoing pandemic, observational designs like interrupted time series provide an opportunity to estimate the association of interventions with observed outcomes to inform subsequent decision-making. Our study has several limitations. First, the data used in this study are from the IMSS HIS, an administrative data source that may contain errors. However, the HIS staff regularly validate the information provided by the IMSS health facilities, securing data completeness and quality. Second, the model does not account for all factors shaping utilization, potentially affecting the utility of model predictions; unmeasured variables such as fluctuation in service demand or in supply chain could bias IRRs for some services if they are confounded in time with the Recovery policy. For instance, shortages in the BCG supply were observed from January 2020 to February 2021, while from March 2021 a steady provision of this vaccine was established, potentially confounding Recovery policy effects. Third, we assumed a uniform effect of the Recovery policy across delegations (immediate in the main analysis and with a 1-month lag in sensitivity analyses); inconsistencies in timing and intensity of the Recovery policy implementation could affect the findings. Finally, we accounted for the burden of the pandemic using the proportion of IMSS beds occupied by COVID-19 patients on average each month; this may not fully capture the multiple ways that the pandemic shapes health service demand, health-seeking behaviour, and health outcomes.

## CONCLUSIONS

The COVID-19 pandemic has led to declines in essential health care around the world. Despite the ongoing pandemic, health systems must design strategies to resume care provision and address backlogs in care. Our results indicate that policies to tackle essential health services recovery need to be informed by local data to adapt to a changing context and target services with slow recovery or backlogs. The present study results could guide IMSS improvement and investment priorities to ensure resources are used for sustainable improvements in the RMCH and NCDs services. Our results may also help other countries facing similar declines in essential health care.

## Additional material


Online Supplementary Document
