# Supplementary material for: The road to recovery: an interrupted time series analysis of policy intervention to restore essential health services in Mexico during the COVID-19 pandemic
Source: J Glob Health. 2022 Jul 23;12:05033. doi: 10.7189/jogh.12.05033 (PMC9304921; doi:10.7189/jogh.12.05033)
Supplement: Online Supplementary Document [file jogh-12-05033-s001.pdf]

Supplementary Document

Table S1. Definitions of the service delivery indicators

Indicator	Numerator	Denominator
**Child health care**		
Number of consultations for sick children	Number of under-5 children visited primary care clinics for sick childcare including for diarrhea, pneumonia or malnutrition	-
Childhood vaccinations	**BCG vaccine** (Number of children vaccinated with a unique dose of BCG)	-
	**Pentavalent vaccine** (Number of children vaccinated with a 3rd dose pentavalent + Number of children vaccinated with a 3rd dose of pentavalent acellular)	-
	**MMR vaccine** (Number of children vaccinated with the second dose of the triple viral measles, mumps and rubella vaccine)	-
	**Pneumococcal vaccine** (Number of children who received the third dose of pneumococcal vaccine)	-
	**Rotavirus vaccine** (Number of children who received the second dose of the rotavirus vaccine)	-
**Reproductive and maternal health care**		
Contraceptive use visits	Number of reproductive-age women who used contraceptive services and accepted a contraceptive prescription for the first time or for a refill	-
Antenatal care visits	Total number of antenatal care visits (including first and subsequent visits)	-
Total facility deliveries	Number of vaginal deliveries + Number of caesarean sections	-
C-section rate %	Number of caesarean sections	Number of vaginal deliveries<br>+ Number of caesarean sections
**Cancer screenings**		
Screened for cervical cancer	Number of women aged 25 - 64 screened with Papanicolaou test for cervical cancer	-
Screened for breast cancer	Number of women aged 50 - 69 screened for breast cancer for the first time with a mammography	-
**Noncommunicable disease care**		
Diabetes visits	Number of diabetic patients visited primary care clinics (20+ years)	-
Hypertension visits	Number of hypertensive patients visited primary care clinics (20+ years)	-
Controlled diabetes %	Number of diabetic patients (20+years) with fasting blood glucose tests 70–130 mg/dl	Number of diabetic patients visited primary care clinics

		(20+years)
Controlled hypertension %	Number of hypertensive patients (20+years) with blood pressure <140/90mmHg	Number of hypertensive patients visited primary care clinics (20+years)

Table S2. Childhood vaccines administered pre- and during COVID-19, Mexican Institute of Social Security, January 2019-November 2021

Vaccine	Pre-COVID-19 period, Jan 2019 – Mar 2020<br>N=35 delegations	COVID-19 period before Recovery policy, Apr 2020 – Mar 2021<br>N=35 delegations		COVID-19 Recovery policy period, Apr 2021 – Nov 2021<br>N=35 delegations	
	Median delegation (Q1, Q3)	Median delegation (Q1, Q3)	% of pre-pandemic level	Median delegation (Q1, Q3)	% of pre-pandemic level
BCG	444 (332, 702)	167 [99, 256)	38%	653 (378, 963)	147%
Pentavalent (3rd dose)	1 133 (761, 1 822)	448 (211, 687)	40%	484 (215, 672)	43%
MMR (2nd dose)	148 (55, 265)	153 (68, 240)	103%	265 (127, 456)	179%
Rotavirus (2nd dose)	314 (170, 652)	258 (121, 465)	82%	211 (150, 508)	67%
Pneumococcal (3rd dose)	83 (52, 192)	52 (33, 141)	63%	69 (39, 145)	83%

BCG: Bacillus-Calmette Guérin. MMR: Measles, Mumps and Rubella.

Table S3. Poisson regression models of Recovery policy on childhood vaccinations April 2020 – November 2021, Mexican Institute of Social Security (N=700 delegation-months)

Vaccine	Level effect of the Recovery policy	Trend effect of the Recovery policy per month
	IRR [95% CI]	IRR [95% CI]
BCG	8.83 [6.75, 11.55]	1.03 [1.00, 1.06]
Pentavalent (3rd dose)	0.93 [0.86, 1.00]	0.98 [0.96, 1.00]
MMR (2nd dose)	1.29 [1.21, 1.38]	0.96 [0.94, 0.99]
Rotavirus (2nd dose)	0.97 [0.89, 1.06]	0.97 [0.95, 0.99]
Pneumococcal (3rd dose)	1.12 [1.04, 1.20]	0.92 [0.90, 0.95]

Poisson models include data from April 2020 to November 2021 (COVID-19 period). Models are adjusted for months since the start of Covid, average number of beds occupied by COVID-19 patients per month (log transformed), and seasonality. Population exposure is defined as the 2021 IMSS population per delegation, limited as follows: children under 1 for BCG, children under 10 for all other vaccinations.

BCG: Bacillus-Calmette Guérin. CI: Confidence interval. IRR: Incidence Rate Ratio. MMR: Measles, mumps and rubella.

Table S4. Alternative models of Recovery policy on health service use and outcomes April 2020 – November 2021, Mexican Institute of Social Security (N=700 delegation-months)

	Linear models		Poisson models with 1 month lag for Recovery policy	
	Level effect of the Recovery policy	Trend effect of the Recovery policy per month	Level effect of the Recovery policy	Trend effect of the Recovery policy per month
Indicator	Beta [95% CI]	Beta [95% CI]	IRR [95% CI]	IRR [95% CI]
**Child healthcare**				
Consultations for sick children	51.11 [36.34, 65.89]	-16.75 [-20.30, -13.20]	1.18 [1.10, 1.26]	0.87 [0.85, 0.89]
Childhood vaccinations	862.85 [477.03,1248.67]	-8.34 [-42.74,26.06]	1.49 [1.27, 1.74]	0.93 [0.91, 0.94]
**Reproductive and maternal healthcare**				
Contraceptive use visits	-20.86 [-71.74, 30.02]	-12.84 [-26.03, 0.36]	1.06 [1.01, 1.11]	0.94 [0.92, 0.96]
Antenatal care visits	96.81 [20.74, 172.88]	-16.96 [-56.44, 22.51]	1.09 [1.06, 1.11]	0.98 [0.98, 0.99]
Deliveries (includes caesarean sections)	83.51 [50.45, 116.58]	28.35 [21.01, 35.70]	1.13 [1.09, 1.17]	1.03 [1.02, 1.04]
Caesarean section deliveries	-0.36 [-1.53, 0.80]	-0.97 [-1.20, -0.73]	1.00 [0.97, 1.02]	0.99 [0.98, 0.99]
**Cancer screening**				
Cervical cancer screening	-280.39 [-504.29, -56.50]	44.84 [-79.66, 169.34]	1.36 [1.23, 1.50]	0.93 [0.91, 0.96]
Breast cancer screening	-82.43 [-227.99, 63.13]	88.21 [29.02, 147.39]	1.51 [1.36, 1.67]	0.94 [0.90, 0.98]
**Noncommunicable disease care**				
Diabetes visits	849.98 [99.22, 1600.74]	274.23 [-128.24, 676.70]	1.10 [1.08, 1.12]	1.02 [1.00, 1.03]
Hypertension	408.36	-1062.85	1.09	0.98

visits	[-354.69, 1171.41]	[-1563.57, -562.13]	[1.06, 1.12]	[0.97, 0.99]
Controlled diabetes	0.61<br>[-0.04, 1.26]	0.92<br>[0.66, 1.17]	1.14<br>[1.07, 1.22]	1.02<br>[1.01, 1.03]
Controlled hypertension	4.47<br>[3.54, 5.41]	0.97<br>[0.67, 1.26]	1.25<br>[1.21, 1.30]	1.00<br>[0.99, 1.00]

Models include data from April 2020 to November 2021 (COVID-19 period). Models are adjusted for months since the start of Covid, average number of beds occupied by COVID-19 patients per month (log transformed), and seasonality. Population exposure for Poisson models is defined as the 2021 IMSS population per delegation, limited as follows: women 15-49 for contraceptive visits, antenatal care visits, deliveries, total deliveries for cesarean deliveries, children under 5 for sick child visits, children under 10 for vaccinations, women 25 to 64 for cervical cancer screening, women 50 to 69 for breast cancer screening, adults over 20 for diabetes and hypertension consultations, and consultations for diabetes or consultations for hypertension for control of each disease. For cesarean section deliveries, controlled diabetes, and controlled hypertension, linear models provide % outcomes. CI: Confidence interval. IRR: Incidence Rate Ratio.
